# Evaluating the use of locally-based health facility assessments in Afghanistan: a pilot study of a novel research method

**DOI:** 10.1186/1752-1505-8-24

**Published:** 2014-11-25

**Authors:** Jack S Rowe, Kayhan Natiq, Olakunle Alonge, Shivam Gupta, Anubhav Agarwal, David H Peters

**Affiliations:** Johns Hopkins Bloomberg School of Public Health, Baltimore, USA; Massachusetts General Hospital, Boston, USA; Indian Institute of Health Management Research, Jaipur, India

**Keywords:** Health services, Public Health, Research methods, Conflict, Afghanistan

## Abstract

**Background:**

Through the Balanced Scorecard program there have been independent, annual and nationwide assessments of the Afghan health system from 2004 to 2013. During this period, Afghanistan remained in a dynamic state of conflict, requiring innovative approaches to health service evaluation in insecure areas. The primary objective of this pilot study was to evaluate the reliability of health facility assessments conducted by a novel, locally-based data collection method compared to a standard survey team.

**Methods:**

In this cross-sectional study, one standard survey team of clinicians and multiple rapidly trained locally-based survey teams of teachers conducted health facility assessments in Badghis province, Afghanistan from March – August, 2010. Outpatient facilities covered under the country’s Basic Package of Health Services were eligible for inclusion. Both approaches attempted to survey as many health facilities as safely possible, up to 25 total facilities per method. Each facility assessed was scored on 23 health services indicators used to evaluate performance in the annual Balanced Scorecard national assessment. For facilities assessed by both survey methods, the indicator scores produced by each method were compared using Spearman’s correlation coefficients and linear regression analysis with generalized estimating equations.

**Results:**

The standard survey team was able to assess 11 facilities; the locally-based approach was able to assess these 11 facilities, as well as 13 additional facilities in areas of greater insecurity. Among the 11 facilities assessed by both approaches, 19 of 23 indicators were statistically similar by survey method (p < .05). Spearman’s coefficients varied widely from (−0.39) to (0.71). The differences were greatest for items requiring specialized data collector knowledge on reviewing patient records, patient examination and counseling, and health worker reported satisfaction.

**Conclusions:**

This pilot study of a novel method of data collection in health facility assessments showed that an approach using locally-based survey teams provided markedly increased access to areas of insecurity. Though analysis was limited by small sample size, indicator scores used for facility evaluation were relatively comparable overall, but less reliable for items requiring clinical knowledge or when asking health worker opinions, suggesting that alternative approaches may be needed to assess these parameters in insecure environments.

## Introduction

The Balanced Scorecard (BSC) has been an essential component of health services monitoring and evaluation in Afghanistan since 2004, when it was created to assess the implementation of the country’s Basic Package of Health Services (BPHS). Since then, the BSC provided a robust assessment of health sector performance throughout the country, assisting policy-makers and managers in identifying and addressing gaps in service provision and quality of care [1–5]. The generation of the BSC relied on highly-trained survey teams of medical professionals to conduct in-depth health facility assessments across the country. BSC scores were then calculated for each facility, which were aggregated to the provincial and national levels [1–3].

Afghanistan has remained in a dynamic state of conflict with unique challenges for data collection and increasing areas of insecurity [6–11]. Standard BSC survey teams, although composed of native Afghans, were often viewed as outsiders when visiting more remote regions of the country. During data collection for the 2008 and 2009/2010 BSCs, randomly selected facilities were replaced with those in more secure locations in 28 and 29 of 34 total provinces, respectively, due to insecurity. Such re-sampling resulted in an absence of essential information about critical areas of the country and potentially introduced selection bias into the BSC provincial and national scores. Due to worsening insecurity in significant parts of Afghanistan, innovative methods for data collection are required that optimize both team safety and data accuracy and ensure that the BSC remained a reliable and representative measure of health system performance. Such methods are also essential to estimate the impact of insecurity on health service delivery and to assess bias introduced by restricting sampling to secure facilities.

There is an expanding literature base on national health systems performance assessment and health system surveys in low and middle income countries [12–17]. However, there is limited literature available on the practical approaches to conducting such assessments in areas of recent or active conflict [12, 18–23]. Locally-based data collection teams are generally viewed as less intrusive and can provide a critical alternative in conducting facility assessments insecure areas. Incorporating local community members as data collectors can also facilitate further engagement between community members, researchers, and national level policy-makers and increase potential engagement with the formal health sector. Teachers have previously been key components of health-related educational campaigns; however, their involvement in health services research is limited. Utilizing teams of local teachers ensures the data collectors are literate and generally available in all areas of the country, regardless of security context [24–26]. While community-based data collection has been used extensively in low- and middle-income countries, we are not aware of any studies comparing health facility assessments between professional data collection teams and rapidly-trained teams of community members [27]. Documentation of surveyor training among health facility assessments in the peer-reviewed and grey literature is often limited and highly variable [12].

The primary objective of this pilot study was to evaluate the ability of rapidly trained locally-based survey teams of primary and secondary school teachers to conduct health facility assessments and to assess the reliability of this data as compared to a standard survey team in Badghis province, Afghanistan. The secondary objective was to compare the locally-based assessment of facilities in secure versus insecure regions of Badghis province. We hypothesized that data collected would be similar between the two survey methods and that indicators of health service provision would be lower in insecure areas. This study addresses questions relevant not only to improve health facility assessments in Afghanistan, but to health systems evaluation in any area of conflict or insecurity.

## Methods

### Study design and site selection

This research was conducted on a method to implement the larger Balanced Scorecard national assessment, which was approved by the Johns Hopkins University and Afghan Ministry of Public Health institutional review boards. Badghis province was chosen for its range of secure and insecure areas. Badghis is a province in western Afghanistan covering 20,068 square kilometers of largely mountainous or semi-mountainous terrain, and it is divided into 7 districts [28]. A United Nations report released in October 2009 classified 1 district in Badghis as “low risk”, 2 districts as “medium risk”, 4 districts as “high risk”, and 0 districts as “very high risk” [11]. In 2010, the Afghanistan NGO Safety Office (ANSO) classified Badghis province as “moderately insecure”, on the scale of “low insecurity”, “deteriorating”, “moderately insecure”, “highly insecure”, or “extremely insecure”, with 356 total reported attacks by armed opposition groups in Badghis in 2010 [29]. The total population in Badghis is estimated to be 499,393 people, with 97% of the population living in rural areas [28].

Facilities eligible for inclusion were those covered under the BPHS package in Badghis: sub-health centers (SHC), basic health centers (BHC), or comprehensive health centers (CHC). District, provincial, and regional hospitals were excluded, since the focus of the BSC assessment is on a basic package of health services at predominantly outpatient-oriented facilities [1, 2, 4]. Of the 40 BPHS facilities in Badghis at the time, a stratified random sample of 25 BPHS facilities selected for assessment was generated, the sample size used to calculate BSC scores in each province. A standard survey team of physicians, nurses, and a pair of monitor-supervisors, upon arriving in Badghis, then met with key provincial officials from the Ministry of Public Health’s Department of Monitoring and Evaluation, Provincial Health Department, and Provincial Educational Department, and other key local stakeholders to determine the security status of facilities selected for sampling. Based on this discussion, the standard team was deemed safely able to assess 11 “secure” facilities; the approach using locally-based teams was able to assess those 11 “secure” as well as 13 additional “insecure” facilities (24 total). Because of the incredibly dynamic security environment in Afghanistan, we chose to use local informants as the guide to the security status, as opposed to using district level security scores, such as those used by various intergovernmental and nongovernmental organizations in Afghanistan [11, 29]. It was felt that relying on these scores might place surveyors at undue risk, as these reports often do not represent the most up to the minute security context, are dependent on the ability to report security incidents (some of the most dangerous areas had few people reporting incidents), and the survey teams placed more faith in informed, local knowledge.

Facility assessments incorporated observation of patient-provider clinical interactions with follow-up exit interviews of the patients, health worker interviews, and facility record audits. Survey instruments contained a mixture of continuous, binary, and categorical variables. Categorical variables were scored using Likert scales. Locally-based teams were trained with abridged survey instruments containing only questions necessary for calculation of the BSC, compared to survey instruments used by the standard team that included a number of research-related questions. For each facility surveyed, observation of patient care was based on a systematic sample of clinical interactions between children and adults with the main health worker, with targets of 5 adult and 5 child patients selected using a random starting point and sampling interval determined by the average number of new patients per day. Following observation of patient-provider clinical interaction, patients were invited for an exit-interview, away from any local health-care providers. A target of 4 health workers were also randomly sampled and selected for interview at each facility, stratified by the type of health worker. One facility record audit was completed for each facility [1, 2, 4].

### Selection and training of locally-based teams

Upon arrival in Badghis, the standard team and pair of monitor-supervisors worked with the Provincial Education Department to identify suitable, documented, and qualified teachers to comprise the locally-based teams. To be selected for a locally-based team, the teacher must have resided in the catchment area of the facility that they would evaluate at the time of the survey and have stated they had had no relationship with the workers at that facility. Teachers were primary or secondary teachers, with preference given to secondary (high school) teachers, who were felt to be more capable at completing complex tasks. Because teachers must come from the catchment area of the facility surveyed, a different locally-based team composed of two teachers was used to survey each facility assessed by that method; whereas, only one standard survey team was used for the entire province.

For each facility to be surveyed by the locally-based method, a pool of three to five teachers who were willing to participate travelled to the provincial capital, where they collectively underwent three days of intensive training. During the training period, the monitor-supervisors gave instruction on ensuring data quality, interviewing techniques, research ethics, and patient selection, and were familiarized with the survey tools to be used. Key medical equipment and aspects of hospital infrastructure were demonstrated. Training culminated in a field testing exercise, followed by a post-training exam to assess understanding of the study protocol. For each facility to be surveyed, the two teachers scoring highest on the post-training exam were retained from the original pool of three to five teachers for that given facility. This rapid training was in contrast to the standard team, which was comprised of Afghan health professionals from throughout the country, most of whom had years of experience in survey data collection. Prior to data collection the standard team underwent an annual, two week training on survey tools and procedures in Kabul that included extensive field testing and post-training exams.

Each of two monitor-supervisors was paid $600 US Dollars (USD)/month as part of their annual contract, in addition to a $15 USD/day per diem for days spent in the field. All four members of the standard survey team received $500 USD/month plus a $15 USD/day per diem while in the field. Each of the 48 locally-based surveyors received $80 USD total for their work on this project.

### Data collection

The standard survey team collected data in Badghis during March-April, 2010; however, due to delays in participant selection and training, locally-based teams were not able to collect data until July-August, 2010. A maximum of 2 days was given to complete each facility assessment. Once finished, locally-based teams returned to the provincial capital to meet with the provincial supervisor, who ensured completion of the survey tools and confirmed the local team’s visit to the facility by phone. Participants on the local teams were reimbursed for their time upon verification of survey completion. During the period of data collection, supervisors conducted active monitoring of the locally-based teams by randomly selecting 2 facilities in secure areas to which they accompanied the survey teams. Post-monitoring was conducted on 4 randomly selected facilities in secure areas, where highly-trained monitors re-surveyed the facility using only the facility record audit survey tool one day after the locally-based teams finished. Upon review of all questions administered at the 4 secure facilities selected for post-monitoring, there was a 91% concordance rate in the data generated by the supervisors and locally-based teams.

### Data analysis

Data were analyzed using STATA version 10 (Stata Corp, College Station, TX). Scales and indices used in the calculation of BSC scores were generated from the survey data for categorical and continuous variables, respectively. Details of BSC indicator composition are discussed elsewhere [1, 2, 4]. Briefly, each of the 23 indicators was generated from 1 to 19 component variables that are included in the BSC facility survey tools. All indicator scores in this study were continuous variables that ranged from 0 (poor) to 1 (excellent).

For the primary objective of assessing the reliability between the locally-based and standard survey methods, only the 11 facilities visited by both survey methods were used to compare 23 BSC indictors. Spearman rank-correlation coefficients were used to compare these indicators by survey method (standard versus locally-based), and chi-squared analysis was performed to assess statistical significance of aggregate demographic data. Because each of the 11 overlapping facilities was assessed once by each survey method and each facility contained multiple observations of health workers and patients, a linear regression model with generalized estimating equations (GEE) and robust variance estimation was used to account for correlations within the repeated measures of the health service indicators at each facility. P-values were generated using GEE regression models to determine the influence of survey method on the given outcome. GEE regression with robust variance estimation has been validated for sample sizes less than 10 [30]. Kappa scores were not used, given that our analysis required comparing multiple data points paired by the individual facilities assessed, instead of a comparison of aggregate, unpaired data.

For the secondary objective of comparing health service provision at secure versus insecure facilities, we compared indicators generated from the locally-based method for 11 secure and 13 insecure facilities, respectively. This was done using multiple linear regression with GEE controlling for facility type (SHC, BHC, CHC) to account for potential confounding.

## Results

### Characteristics of health facilities, patients, and health workers

A summary of the number and type of facilities assessed, and patients and health workers interviewed by each method is illustrated in Table 1. Locally-based and standard survey teams were able to assess 24 (96%) and 11 (44%) of the targeted 25 facilities, respectively. Based on the UN security classification system used at the time, the standard team was able to assess 4 facilities in the “low risk” district, 3 facilities in the “medium risk” districts, and 4 facilities in the “high risk district”. The locally-based method was able to assess these facilities, as well as an additional 2 facilities in the “medium risk” districts, and 11 other facilities in the “high risk” districts [11]. Table 2 illustrates the demographic data for patients who were observed and interviewed and for health workers interviewed among the 11 facilities assessed by both survey methods. For these 11 facilities, our survey teams engaged with 203 patients. There was no significant difference in the age or sex of patients observed and interviewed, by survey method (p = 0.70). There was no significant difference in the types of health workers interviewed by survey method (p = 0.95); both were most likely to interview vaccinators.

**Table 1:** **Summary of sample according to method of data collection**

	Standard method	Locally-based method	Total
**Period of facility assessments**	March-April, 2010	July-August, 2010	March-August, 2010
**Number of survey teams used**	1	24	25
**All unique facilities assessed**	11	24	24
**Sub-Health Center**	2	6	6
**Basic Health Centers**	8	16	16
**Comprehensive Health Centers**	1	2	2
**All patients observed/interviewed**	94	216	310
**All health workers interviewed**	30	64	94

**Table 2:** **Characteristics of patients and health workers among facilities assessed by both standard and locally-based methods**

	All [n(%)]	Standard method [n(%)]	Locally-based method [n(%)]	P-value ^1^
**All patients**	203 (100)	94 (100)	109 (100)	0.70
**<5 years old**	98 (48)	44 (47)	54 (50)	
**≥5 years old**	105 (52)	50 (53)	55 (50)	
**Female**	106 (52)	53 (56)	53 (49)	
**Male**	95 (47)	39 (42)	56 (51)	
**Sex unknown**	2 (1)	2 (2)	0 (0)	
**All health workers**	63 (100)	30 (100)	33 (100)	0.95
**Doctor**	5 (8)	2 (7)	3 (9)	
**Assistant doctor**	3 (5)	2 (7)	1 (3)	
**Nurse**	14 (22)	6 (20)	8 (24)	
**Midwife**	14 (22)	7 (23)	7 (21)	
**Vaccinator**	27 (43)	13 (43)	14 (42)	
**Female**	19 (30)	10 (33)	9 (27)	
**Male**	44 (70)	20 (66)	24 (73)	

^1^p-value calculated using chi-squared analysis.

### Comparability of facility scores, by survey method

The mean scores for all 11 facilities assessed by both survey methods are grouped by instrument of data collection and listed in Table 3. Four of the 23 indicators differed significantly by survey method (p-value <0.05): *Patient records, patient counseling, appropriate exam duration,* and *health worker satisfaction*. Notably, 3 of these 4 indicators were calculated from survey data collected via observation of patient-provider interaction. Also among the indicators calculated from patient-provider observation data, standard-method mean scores were uniformly lower than locally-based method scores. Scores generated from locally-based data collection differed by more than 20% of the standard team score for only 3 of 23 indicators: *Patient records, patient counseling,* and *tuberculosis records*. Values for the Spearman’s coefficient ranged widely by indicator, from (−0.39) to (1.0), with 6 of 19 greater than 0.5; however, their interpretation was drastically limited by the small sample size of 11 facilities.

**Table 3:** **Comparison of health service evaluation indicator scores generated from facilities assessed by both standard and locally-based survey methods**

Indicator label	Standard method [mean(SD)]	Locally-based method [mean(SD)]	Spearman’s correlation coefficient	P-value ^1^
**Based on patient-provider observation:**				
**Patient record**	0.48 (0.21)	0.75 (0.25)	−0.08	**0.007**
**Patient history & physical exam**	0.69 (0.13)	0.71 (0.29)	0.18	0.81
**Patient counseling**	0.13 (0.12)	0.50 (0.22)	−0.13	**<0.001**
**Appropriate exam duration**	0 (0)	0.26 (0.44)	Unable to calculate	**0.01**
**Based on patient exit interview:**				
**Patient satisfaction**	0.75 (0.20)	0.76 (0.30)	−0.12	0.90
**Patient perceptions of quality**	0.73 (0.10)	0.67 (0.22)	−0.25	0.32
**Based on health worker interview:**				
**Health worker satisfaction**	0.63 (0.11)	0.70 (0.084)	0.46	**0.02**
**Salary payment current**	0.40 (0.50)	0.27 (0.45)	0.50	0.37
**Provider knowledge: vaccination**	0.72 (0.34)	0.83 (0.27)	−0.39	0.29
**Provider knowledge: integrated management of childhood illness**	0.52 (0.28)	0.51 (0.22)	0.41	0.92
**Provider knowledge: reproductive health**	0.63 (0.22)	0.56 (0.22)	0.31	0.70
**Based on facility record audit:**				
**Drug availability**	0.76 (0.25)	0.93 (0.19)	0.32	0.07
**Family planning availability**	0.82 (0.24)	0.84 (0.17)	−0.27	0.86
**Health management information systems use**	0.70 (0.41)	0.70 (0.32)	−0.06	1.00
**Clinical guidelines**	0.76 (0.28)	0.73 (0.24)	0.71	0.55
**General infrastructure**	0.79 (0.19)	0.70 (0.26)	0.55	0.23
**Proper sharps disposal**	1.0 (0)	0.82 (0.41)	Unable to calculate	0.18
**Outpatient service utilization**	0.88 (0.35)	0.88 (0.35)	1.0	Unable to calculate
**Facilities providing antenatal care**	0.73 (0.47)	0.73 (0.47)	0.54	1.00
**Delivery care according to national guidelines**	0.73 (0.47)	0.82 (0.41)	0.24	0.60
**Females as proportion of new outpatients**	0.57 (0.51)	0.58 (0.78)	0.68	0.64
**Service utilization**	2988 (1480)	3019 (1960)	0.58	0.95
**Tuberculosis register**	0.36 (0.51)	0.55 (0.52)	0.31	0.35

Mean scores represent the average score for a given indicator, among the 11 facilities surveyed by each method; scores ranged from 0 (poor) to 1 (excellent). Among variables used to calculate the indicator scores, 22 of 3866 (0.59%) and 46 of 4312 (1.7%) observations were missing for the standard and locally-based approaches, respectively.

SD = Standard Deviation.

^1^p-value calculated using multiple linear GEE regression comparing indicator scores by survey method; p-values <0.05 are in bold.

### Comparability of facility scores, by security status

The mean scores for both the 11 secure and 13 insecure facilities surveyed by the locally-based approach are listed in Table 4. The four indicators that were found to be significantly different by survey method in Table 3 were thus felt to be unreliable and are not presented in the analysis by security setting in Table 4. Four of the 19 indicators differed by security status (p-value <0.05). *Patient history and physical exam* and *patient perceptions of quality* indicators scored higher in insecure areas, whereas *delivery of care according to national guidelines* and *service utilization* indicators scored lower.

**Table 4:** **Comparison of health service evaluation indicator scores generated from locally-based assessments at secure and insecure facilities**

Indicator label	Secure facilities [mean(SD)]	Insecure facilities [mean(SD)]	P-value ^1^
**Based on patient-provider observation:**			
**Patient history and physical exam**	0.71 (.29)	0.88 (0.17)	**<0.001**
**Based on patient exit interview:**			
**Patient satisfaction**	0.76 (0.30)	0.81 (0.26)	0.26
**Patient perceptions of quality**	0.67 (0.22)	0.74 (0.15)	**0.005**
**Based on health worker interview:**			
**Salary payment current**	0.27 (0.45)	0.26 (0.45)	0.85
**Provider knowledge: vaccination**	0.83 (0.27)	0.68 (0.36)	0.07
**Provider knowledge: integrated management of childhood illness**	0.51 (0.22)	0.40 (0.14)	0.12
**Provider knowledge: reproductive health**	0.56 (0.22)	0.67 (0.078)	0.30
**Based on facility record audit:**			
**Drug availability**	0.93 (0.18)	0.88 (0.25)	0.65
**Family planning availability**	0.84 (0.17)	0.83 (0.24)	0.92
**Health management information systems use**	0.70 (0.31)	0.64 (0.39)	0.67
**Clinical guidelines**	0.73 (0.23)	0.56 (0.28)	0.17
**General infrastructure**	0.70 (0.26)	0.64 (0.27)	0.79
**Proper sharps disposal**	0.82 (0.41)	0.85 (0.38)	0.75
**Outpatient service utilization**	0.88 (0.35)	0.63 (0.52)	0.28
**Facilities providing antenatal care**	0.73 (0.47)	0.54 (0.52)	0.40
**Delivery care according to national guidelines**	0.82 (0.41)	0.39 (0.51)	**0.03**
**Females as proportion of new outpatients**	0.58 (0.078)	0.56 (0.13)	0.65
**Service utilization**	3018 (1960)	1494 (1024)	**0.04**
**Tuberculosis register**	0.55 (0.52)	0.53 (0.52)	0.71

Mean scores represent the average score for that indicator among all facilities of a given security status (11 secure facilities versus 13 insecure facilities). Only facilities that were surveyed by the locally-based approach are included above. Scores ranged from 0 (poor) to 1 (excellent). Among variables used to calculate the indicator scores, 46 of 4312 (1.7%) and 81 of 4312 (1.9%) observations were missing for the secure and insecure facilities, respectively.

SD = Standard Deviation.

^1^p-value calculated using multiple linear regression to compare indicator scores by security level, controlling for facility type (SHC, BHC, CHC); p-values <0.05 are in bold.

### Comparison of cost, by survey method

Total costs of data collection for all facilities surveyed by the standard and locally-based approaches in Badghis were estimated to be $4750 USD and $6240 USD, respectively. This included training costs for the locally-based teams. Given that the standard and locally-based approaches were able to assess 11 and 24 facilities, respectively, the cost per facility surveyed was $432 USD and $260 USD for the standard and locally-based methods, respectively.

## Discussion

Rapidly trained, locally-based teams of teachers were able to conduct complex health facility assessments in areas too insecure for a professional team of experienced surveyors. The locally-based approach was able to assess more than twice the total number of facilities compared to the standard survey method, providing a critical method for gaining insight into the health infrastructure in these insecure areas. In the 11 secure facilities surveyed by both methods, only 4 of 23 indicators had a statistically significant difference between survey methods, indicating that the two methods were relatively comparable in the scores of health system performance they generated.

Some variability in the re-application these comprehensive survey tools is similarly seen in all BSC active-and post-monitoring throughout the country, when trained monitors reassess standard teams both in real-time and within 1 week of survey completion. Due to delays in community-member selection and training, the locally-based survey teams’ assessments came 4 to 5 months after those of the standard-survey team. Because of this, all patients and some of the health workers interviewed were different between each survey method, which inherently introduces additional variability into the generated BSC scores. In Afghanistan, climate, funding, security constraints, available resources, patient demand, and provider availability are often characterized by significant temporal variation, which can influence the BSC scores [6, 8, 9]. The high concordance rate of 91% between monitors and locally-based teams seen in post-monitoring of selected secure facilities supports the validity of the locally-generated data and points to factors other than the type of data collector in accounting for any differences in indicator scores. Notably, insecurity in the country generally peaks in the months when the locally-based teams conducted their assessments, a testament to the ability of locally-based method to access the more insecure areas of the province [31].

Given that 3 of the 4 indicators based on observation of patient-provider interactions were statistically different, locally-based teams may require more intensive direction on elements of the patient encounter and other clinical variables during training. This is reasonable given that, although simplified and standardized, scoring patient-provider interactions requires the most relative clinical acumen and interpretation of clinical activities, compared to the other modalities of data collection.

There was a significant difference in the *health worker satisfaction index* between survey methods (p = 0.02), with the standard team generating a mean score lower than the locally-based teams. The mean *patient satisfaction index* score was also lower in the standard-method group, although not significantly. This may be due to health workers and patients being more willing to share negative opinions with those perceived as outsiders, as compared to members of the same community. Notably, questions related to health worker satisfaction were asked directly by the data collector. In subsequent rounds of BSC data collection, such questions were self-administered to mitigate any associated reporting bias.

When comparing the 11 secure facilities and 13 insecure facilities assessed by locally-based teams, indicator scores were generally lower in areas of insecurity, with *delivery of care according to national guidelines* and *service utilization* markedly lower in insecure areas, even while controlling for the type of facility assessed. These indicate that those working in areas of insecurity may be less able to access training materials, receive proper supervision, or practice an overall standard of care in accordance with national guidelines. Patient ability to access health services may also be limited, resulting in decreased service utilization. However, indicators of *patient history and physical exam* and *patient perceptions of quality* were higher in areas of insecurity. These data indicate that any national health service assessment that is unable to sample facilities in insecure areas likely generates a biased assessment of the province, further highlighting the importance of developing methods for data collection in insecure areas.

The study has several limitations. The small sample size of this pilot study limited the interpretability of the analysis. Only 11 facilities could be assessed by both methods; due to security constraints, the standard team was unable to access more facilities in Badghis province, limiting the power to detect statistically significant differences. Given that indicator scores were continuous, linear GEE regression was used to assess comparability; however, the distribution of the data at times violated the normality assumption of the linear model. While Spearman’s coefficient does not assume normality, its utility is limited when the sample size is less than 10 units, compared to our sample size of 11 facilities for the primary objective [32]. For the secondary objective of examining the effect of insecurity, the sample size was increased to 24 facilities, which increased the strength of statistical comparability. If the locally-based approach is expanded to larger provinces, teams travelling to and from more remote areas may also require additional compensation, given their longer journey to the provincial capital for training and then again to drop off the completed survey forms. This would increase the cost of this approach. The demographic, geographic, and environmental, and security contexts of Afghanistan are also highly diverse; as this study was limited to one province, both in-country and external generalizability may be limited [6, 8, 9, 31].

## Conclusions

We report that this novel approach using rapidly trained teams of locally-based teachers to conduct health facility assessments was able to access far more health facilities than the standard survey team for significantly lower cost per facility surveyed, offering a new method for conducting health systems surveys in areas of conflict. Among facilities surveyed by both methods, the results were relatively similar across most indicators, with statistically significant differences for those requiring more specialized medical knowledge or where there is more potential bias in the responses from health workers because the data collectors are known in their communities. Indicators of health service provision were generally lower in insecure areas, with a marked decrease in service utilization and adherence to national guidelines. Future research is needed to further characterize and optimize the use of a locally-based approach to data collection in health facility assessments in insecure areas.

## Abbreviations

ANSO: Afghanistan NGO Safety Office
BHC: Basic Health Center
BPHS: Basic Package of Health Services
BSC: Balanced Scorecard
CHC: Comprehensive Health Center
GEE: Generalized Estimating Equations
SD: Standard Deviation
SHC: Sub-Health Center
USD: United States Dollar.
